# Artificial Intelligence in Parenteral Nutrition: Enhancing Patient Outcomes Through Global Experience and the Bulgarian Context

**DOI:** 10.3390/nu18060920

**Published:** 2026-03-14

**Authors:** Mariya Koleva, Nikolina Shishmanova, Petya Georgieva, Stanislava Georgieva, Mariya Ivanova

**Affiliations:** Faculty of Pharmacy, Medical University of Varna, 9000 Varna, Bulgaria; maria.koleva@mu-varna.bg (M.K.); nikolina_shishmanova@abv.bg (N.S.); petia.orlinova@gmail.com (P.G.); stassy.s@abv.bg (S.G.)

**Keywords:** digital health, artificial intelligent, machine learning, nutrition, parenteral nutrition, wearables

## Abstract

Artificial intelligence (AI) has shown substantial potential to improve patient outcomes in parenteral nutrition by enabling individualised nutritional strategies, early prediction of metabolic and infectious complications, and optimised real-time clinical decision-making. Evidence from global clinical practice demonstrates that AI integration can enhance patient safety, reduce complication rates, and improve resource utilisation. In Bulgaria, recent developments in parenteral nutrition reflect progress toward standardisation, wider availability of modern formulations, and alignment with international clinical guidelines. However, the adoption of AI-driven systems for personalised nutrition planning and continuous risk assessment remains limited. Key barriers include the availability and quality of clinical data, regulatory and ethical considerations, and the need for targeted training of healthcare professionals. This review highlights both the opportunities and challenges associated with implementing AI in parenteral nutrition in the Bulgarian context. Potential benefits include improved patient outcomes, shorter hospital stays, more efficient healthcare delivery, and alignment with international best practices. At the same time, overcoming infrastructural, regulatory, and educational barriers is essential for successful implementation. Conclusions: The integration of AI into parenteral nutrition requires a multidisciplinary approach that combines clinical expertise, technological innovation, and supportive health policy. Such an approach offers the potential to sustainably enhance patient care in Bulgaria and position national practice in line with leading global standards.

## 1. Introduction

Parenteral nutrition (PN) is a life-saving option when enteral nutrition is not possible, but it carries risks of complications, including infections and metabolic disturbances [1,2].

PN has been widely used in pediatric and adult patients whenever oral or enteral nutrition (EN) is not possible, insufficient or contraindicated [3].

Nutritional needs differ considerably across age groups, especially in neonatal and pediatric populations. Recent ESPGHAN guidelines recommend that newborns have a water intake of 70–80 mL/kg/day, an energy intake of approximately 120 kcal/kg/day, and a minimum protein intake of 2.5–3 g/kg/day [4]. Glucose infusion rates of 3–5 mg/kg/min are typically suggested, but very low birth weight (VLBW) and extremely low birth weight (ELBW) preterm infants may need up to 12 mg/kg/min. Lipid emulsions can be started right after birth for preterm infants at doses of 0.5–1 g/kg/day. Despite these guidelines, optimising nutrition in extremely preterm infants remains difficult due to the complexities of daily clinical management [4].

Daily energy and nutritional requirements across different age groups are calculated using validated formulas and methodologies, taking into account the patient’s individual characteristics and specific clinical condition [2,3,5,6].

Parenteral nutrition is an expensive therapy and is associated with a wide range of complications. PN is considered a high-alert medication; therefore, its use requires policies, systems, and practices focused on safety to minimise patient risk [1]. The most frequent complications are metabolic, including disturbances in glucose, lipid, and electrolyte metabolism such as hyperglycaemia, hypertriglyceridaemia, and electrolyte imbalances, which may occur at any stage of PN therapy [1]. Catheter-related bloodstream infections remain one of the most serious and common complications. Long-term PN, particularly in the context of home parenteral nutrition, is associated with hepatobiliary disorders and metabolic bone disease [7]. To minimise these risks and improve patient outcomes, continuous monitoring and early prediction of metabolic and infectious complications are essential [7].

In current clinical practice, commercially available, factory-prepared PN bags are widely used. However, many patients require individualised nutritional regimens, necessitating the preparation of customised PN solutions tailored to their specific metabolic and clinical needs. These individualised formulations may be prepared in hospital pharmacy settings or achieved using multi-chamber commercial PN bags with adaptable nutrient compositions.

Personalised approaches to parenteral nutrition require systematic analysis of clinical, laboratory, and anthropometric data to optimise the composition of macro- and micronutrients in accordance with individual patient needs. To prevent adverse events and improve patient outcomes—including reductions in complication rates, length of hospital stay, and mortality—precise dosing and dynamic adjustment of parenteral nutrition formulations are essential.

In this context, integrating innovative technologies, including artificial intelligence and machine learning algorithms, offers new opportunities for risk prediction, individualised nutritional planning, and real-time optimisation of parenteral nutrition therapy, ultimately improving the quality of care and clinical outcomes for patients receiving parenteral nutrition.

### Objectives

The primary aim of this review is to analyse the opportunities and challenges associated with implementing artificial intelligence in the management of patients receiving parenteral nutrition in Bulgaria.

Specifically, this review addresses the following research questions:

- How can artificial intelligence and machine learning methods support the optimisation and individualisation of parenteral nutrition therapy?
- What are the potential benefits of AI-based approaches for predicting metabolic and infectious complications associated with parenteral nutrition?
- What challenges and limitations may arise when implementing AI-based systems in clinical practice, particularly in the Bulgarian healthcare context?

## 2. Materials and Methods

This narrative review was conducted through a structured search of multiple scientific databases, including PubMed, Google Scholar, EBSCO, MDPI, Cochrane Library, SpringerLink, and ScienceDirect. The primary focus was on literature related to parenteral nutrition, with emphasis on regulatory frameworks, patient-centered care, reimbursement mechanisms, and the emerging role of artificial intelligence (AI) in supporting nutritional therapy. Search terms included combinations of the following keywords: “parenteral nutrition,” “intravenous nutrition,” “enteral vs. parenteral nutrition,” “National Health Insurance Fund,” “reimbursement,” “artificial intelligence,” “clinical decision support,” and “healthcare data management.” Boolean operators (AND, OR) were used to refine the search.

Inclusion criteria:

- Publications from 2020 to 2025
- Articles written in English or Bulgarian
- Studies containing clinical, preclinical, or policy-related data on parenteral nutrition
- Research addressing regulatory frameworks, reimbursement, or AI applications in healthcare

Exclusion criteria:

- Studies focused solely on enteral nutrition without relation to parenteral therapy
- Publications outside the defined timeframe or in languages other than English/Bulgarian
- Irrelevant topics not directly connected to parenteral nutrition, reimbursement, or AI

A total of 14 articles meeting all inclusion criteria were included. All articles were reviewed by team members and incorporated into the analysis presentation.

To support various statements and identify existing gaps in the field, articles that did not meet all predefined criteria were also included.

**Additional Data Sources**

The review also includes an analysis of national clinical guidelines and regulations approved by the National Health Insurance Fund (NHIF) in Bulgaria concerning parenteral nutrition. Publicly accessible NHIF data were additionally reviewed to assess reimbursement patterns, coverage criteria, and practical implementation in outpatient and inpatient care during the period 1 January 2025 to 31 March 2025.

**Limitations**

Due to limited availability of recent and specific data, particularly regarding AI applications in parenteral nutrition management, historical and archival sources—including policy documents and clinical guidelines dating back to earlier decades—were consulted to support the review. These sources were critically assessed for relevance, credibility, and applicability to modern healthcare contexts. There is a lack of studies focusing on the economic impacts and resource allocation necessary for implementing AI in PN, especially in resource-limited settings like Bulgaria.

## 3. Results

### 3.1. Types of Parenteral Nutrition

The main types of PN utilised in clinical settings are total parenteral nutrition (TPN), partial parenteral nutrition, and cyclic parenteral nutrition, each tailored to the specific clinical and metabolic needs of the patient [6,8,9]. Advances in formulation, delivery methods, and monitoring technologies have significantly improved the safety and efficacy of PN, helping to mitigate complications historically associated with its use.

#### 3.1.1. Total Parenteral Nutrition (TPN)

TPN provides complete intravenous nutritional support, delivering all essential macronutrients and micronutrients when gastrointestinal function is severely compromised [6]. Due to its hypertonic nature, TPN is typically administered via a central venous catheter, which allows for higher concentrations of nutrients to be safely infused [6]. Clinical studies have demonstrated that TPN improves nutritional status and body weight in patients, particularly in high-risk populations such as those undergoing bone marrow transplantation [8].

#### 3.1.2. Partial Parenteral Nutrition

Partial PN involves the supplementation of oral or enteral intake with parenteral nutrients, offering a balanced approach for patients who can tolerate some gastrointestinal nutrition [6,8,9]. This method is particularly useful in preventing malnutrition in patients with partial GI function, but it requires careful monitoring, as it may be associated with complications such as reductions in visceral protein levels [8,10].

#### 3.1.3. Cyclic Parenteral Nutrition

Cyclic Parenteral Nutrition (PN) delivers nutrients over a set period, usually at night, giving patients more freedom and comfort during the day [6]. This method benefits long-term PN patients by potentially reducing metabolic issues associated with continuous infusion and enabling customisation based on individual metabolic requirements [6,8]. Additionally, cyclic PN has been shown to improve blood sugar regulation and liver health [1,8] in certain patient groups [8,10].

### 3.2. Complications

While parenteral nutrition (PN) is a life-saving therapy for patients unable to receive adequate enteral nutrition, it is not without risks. The most commonly observed complications can be grouped into metabolic, infectious, and long-term categories, arising from the composition of nutritional solutions, the methods of administration, and the duration of therapy [1,11,12].

Metabolic complications are among the most frequent and can occur at any stage of PN therapy. Patients may experience hyperglycemia, hypertriglyceridemia, or electrolyte imbalances, all of which require careful monitoring [1]. Overfeeding, as with underfeeding, poses a significant risk; excessive caloric intake can precipitate a range of metabolic disturbances, including fatty liver, hypercapnia, and fluid overload [6]. Prolonged PN use may also contribute to hepatobiliary and bone disorders, particularly in patients receiving home-based PN, underscoring the need for ongoing assessment [1,13].

Infectious complications primarily stem from the need for venous access. Central venous catheters, commonly used for PN, can predispose patients to catheter-related bloodstream infections, thrombosis, and other vascular complications [1,13,14]. The risk of sepsis is particularly heightened in individuals receiving total parenteral nutrition if strict aseptic technique and catheter care are not maintained [12].

Long-term complications arise with prolonged PN therapy. Chronic exposure to parenteral solutions may cause metabolic or lactic acidosis, with neonates and other vulnerable populations being particularly susceptible [12]. Additionally, prolonged PN use carries the risk of cumulative damage to the kidneys, liver, and biliary system, emphasising the importance of routine monitoring and proactive management [1,8].

PN therapy, while essential for patients unable to utilise the gastrointestinal tract, carries the potential for metabolic, infectious, and long-term complications. Careful planning, vigilant monitoring, and individualised adjustments of PN therapy are crucial strategies to minimise these risks and optimise patient outcomes [1,6,12].

### 3.3. Artificial Intelligence in Parenteral Nutrition

Artificial intelligence (AI) has demonstrated significant potential to anticipate complications associated with parenteral and enteral nutrition. For example, AI-based tools can identify patients at risk of enteral feeding intolerance or refeeding hypophosphataemia, enabling clinicians to implement preventive measures [15]. Machine learning algorithms further enhance personalised care by analysing large, complex clinical datasets to detect patterns and predict which patients are most likely to experience adverse events, thereby supporting targeted and timely interventions [16].

Artificial intelligence offers transformative potential by enhancing the accuracy, safety, and personalisation of parenteral nutrition regimens [17]. Specifically, AI technologies, such as machine learning and natural language processing, can analyse large datasets to predict nutritional outcomes, identify risk factors, including malnutrition and central line-associated bloodstream infections, and provide real-time, evidence-based insights for healthcare professionals [17]. This integration enables a proactive approach to patient management, moving beyond conventional methods of nutritional assessment and intervention. The most advanced technological developments in parenteral nutrition are observed in total parenteral nutrition (TPN), where maximal precision and safety of therapy are required.

AI-driven decision support tools can streamline the prescription process for total parenteral nutrition by formulating standardised regimens and recommending the most suitable option based on individual patient characteristics, thereby reducing human error and resource allocation [16]. Such advanced computational methods enable rapid analysis of complex patient data, including laboratory results, medication records, and clinical assessments, which can then be used to create highly individualised nutrition plans that adapt to dynamic patient needs [17].

This level of personalisation, driven by AI, extends to optimising nutrient delivery and dosage, minimising complications, and ultimately improving patient outcomes through continuous refinement of nutritional support strategies [12]. One notable application of AI in this domain is the development of systems that use AI models to generate standardised parenteral nutrition formulas, thereby enhancing safety and cost-efficiency while remaining responsive to dynamic patient requirements [15]. The application of AI in this field extends beyond mere prescription generation, delving into predictive modelling of lipid emulsion stability, a critical safety concern in parenteral nutrition, to quantify the influence of various determinants [16].

Artificial intelligence (AI)-based systems for parenteral nutrition (PN) are grounded in established clinical nutrition principles and traditional predictive equations, such as the Harris–Benedict formula for estimating energy expenditure and guideline-recommended protein targets. These baseline calculations are continually refined by integrating dynamic, patient-specific data, including laboratory parameters (e.g., electrolytes, glucose levels), vital signs, organ function indicators, and concurrent pharmacological therapies [6].

#### 3.3.1. Machine Learning in Nutritional Prediction

Machine learning techniques, including random forest and extreme gradient boosting, have shown considerable promise in predicting nutritional needs and postnatal growth failure in neonatal patients. Studies report strong performance metrics, such as high AUROC values, demonstrating the reliability of these models in clinical practice [18,19]. By leveraging large clinical datasets, AI models can accurately estimate macronutrient requirements and predict weight changes in infants, offering a level of precision that supports individualised nutritional care [20,21,22]. Artificial intelligence has been successfully utilised to monitor and predict the stability of lipid emulsions in parenteral nutrition formulations, contributing to improved safety and quality control [17].

#### 3.3.2. Standardisation of Nutritional Protocols

The introduction of systems such as TPN2.0 has transformed total parenteral nutrition by standardising formulas based on extensive historical and clinical data. This approach not only improves patient safety but also helps reduce the costs associated with PN preparation [20]. AI-driven decision support tools further streamline the nutritional planning process, reducing reliance on subjective judgement and promoting adherence to evidence-based guidelines [22].

#### 3.3.3. AI in the Management of Complications in Parenteral Nutrition

Recent advances in artificial intelligence have enabled the development of predictive models and decision-support systems that may assist clinicians in managing the risks associated with parenteral nutrition. These technologies can analyse complex clinical variables and help identify patients at increased risk of complications. Table 1 summarises key studies investigating the role of artificial intelligence in the monitoring and management of parenteral nutrition-related complications.

#### 3.3.4. Integration of Clinical Data

AI also facilitates the seamless integration of nutrition-related information from electronic health records, enabling real-time analysis that informs clinical decisions [22]. By combining AI with comprehensive clinical datasets, predictive models have been developed to help clinicians identify patients at higher risk of complications and tailor nutritional interventions accordingly [19]. This integration enhances the precision and responsiveness of nutritional care, ensuring that interventions are both timely and individualised.

By processing large volumes of real-time and longitudinal clinical data, AI algorithms can identify metabolic trends and predict nutrition-related complications, including refeeding syndrome–associated hypophosphataemia, electrolyte imbalances, and disturbances in glucose homeostasis [26]. This predictive capacity enables early intervention and proactive modification of PN prescriptions before clinically significant adverse events occur.

AI systems help optimise PN composition by regularly adjusting amino acid, glucose, and lipid emulsion levels in response to the patient’s evolving metabolic tolerance and clinical condition [27]. Continuous monitoring and adjustments enhance the precision and safety of PN administration, enabling more personalised and adaptive nutritional treatment [23]. Table 2 outlines the main uses of artificial intelligence in parenteral nutrition.

Functional Artificial Intelligence for Parenteral Nutrition is an innovative model based on transformer architectures that optimises and standardises total parenteral nutrition (TPN) by leveraging routinely collected data from electronic health records (EHRs) [20,22]. By integrating complex, multifactorial data—including age, body weight, body mass index (BMI), physical activity level, laboratory parameters (such as serum albumin and C-reactive protein), and baseline energy requirements—the model provides highly accurate predictions of nutritional needs.

Furthermore, clinical AI for Parenteral Nutrition has the potential to incorporate dynamic clinical variables, such as sepsis, acute respiratory distress syndrome (ARDS), or multi-organ failure, enabling real-time adjustment of parenteral or enteral nutrition support [29]. This level of personalisation could minimise the risk of underfeeding or overfeeding, reduce the length of hospital stay, and improve overall clinical outcomes. The model has also been shown to reduce medical errors by up to 50%. Additional potential applications include integration with existing EHR systems to facilitate automated, dynamic nutritional assessment. However, further clinical studies are needed to validate the model in real-world hospital settings and to assess its effectiveness compared with standard nutritional therapy approaches.

PN in hospital settings requires specialised medical equipment that ensures the safe, controlled, and sterile delivery of nutrients directly into the patient’s bloodstream.

The volumetric infusion pump (Volumetric Infusion Pump), also known as an intravenous pump or infusion pump, is a device used to deliver fluids, medications, or nutrients into the patient’s bloodstream at a controlled rate. These pumps are commonly used in hospitals, clinics, and other healthcare facilities. The pump can be programmed to control the infusion rate, volume, and duration. This allows healthcare professionals to administer an exact amount of fluids or medication according to the patient’s needs.

The pump enables precise control of the infusion rate, usually measured in millilitres per hour (mL/h) or drops per minute (gtts/min). This ensures that medications or fluids are delivered at a constant and controlled rate. It can also be set to deliver a predefined volume of fluid or medication. Once the programmed volume has been infused, the pump either stops automatically or activates an alarm system.

Alarm systems are built-in safety features that alert healthcare professionals if a problem occurs during infusion, such as occlusion (blockage) of the infusion line, low battery, or completion of the infusion. Some pumps are equipped with a bolus mode, which enables rapid delivery of a larger volume of fluid or medication over a short period. This function is commonly used in emergency situations or when a higher dose is required.

Mixing devices and automated compounders are used for the preparation of TPN solutions in hospital pharmacies with high accuracy [30]. They automate the dosing and mixing of components, reducing human error and the risk of contamination. They allow precise mixing of nutrients with minimal staff interaction with the preparation. Examples of computerised systems for formulation (prescription) development and verification include software that checks ingredient compatibility, caloric balance, and allergies (e.g., TPN Manager, Neptune Decision Support System). These systems can be integrated with hospital information systems (EHRs) to monitor therapy. A comparison between pumps and compounders is presented in Table 3.

The balance between automation and human oversight is essential for achieving optimal outcomes in clinical nutrition. The quality, accessibility, and standardisation of data are critical factors for the successful implementation of artificial intelligence (AI) in practice. AI is expected to transform clinical nutrition by enabling more precise, personalised, and dynamic therapeutic strategies. These technologies can support early diagnosis, risk prediction, and real-time adaptation of nutritional regimens in critically ill patients. By analyzing large volumes of medical data, AI can provide clinicians with rapid and accurate recommendations, while machine learning models can tailor the composition and dosing of parenteral nutrition (PN) to the individual needs of each patient. Systems with AI for Parenteral Nutrition offer solutions that approach expert-level decision-making. The integration of AI into PN offers multiple benefits, including improved safety and efficacy of therapy, reduced risk of complications, optimised use of resources and clinician time, and the ability to dynamically adjust nutrient delivery according to the patient’s evolving condition.

#### 3.3.5. Achievements in Parenteral Nutrition in Bulgaria

In Bulgaria, ready-to-use parenteral nutrition products registered as medicinal products are mainly used. They are regulated by law [30]. In some hospital departments, parenteral solutions prepared in hospital pharmacies are also used, customised to the patient’s needs in line with prescribed formulations. The National Health Insurance Fund reimburses parenteral solutions in line with clinical pathways [3]. Bulgaria has established clear objectives for healthcare digitalisation and is developing health technology assessment programs to support the funding and adoption of innovations in healthcare [31]. This steers the country toward adopting innovative solutions to enhance patient outcomes. The implementation of artificial intelligence in healthcare requires the adoption of regulatory and technological strategies to protect patient data and adequately support the healthcare process [34]. Currently, regulatory measures are being introduced at the European level, but the precise regulations are still being refined [34].

The use of AI and ML in medicine in Bulgaria is primarily developing within research centres, universities, and some specialised hospitals [35].

In Bulgaria, parenteral solutions, including TPH, are prepared at Alexandrovska Hospital (Sofia); St. Ivan Rilski University Hospital (Sofia); Pirogov University Hospital (Sofia), St. Georgi University Hospital (Plovdiv), St. Marina University Hospital—Varna. Most medical institutions use manual or partially computerised processes for their preparation in classic clean rooms—sterile boxes in their adjacent hospital pharmacies (Aseptic preparation unit). The conditions under which the solutions are prepared include: class A laminar flow cabinet; class A or C GMP clean room; trained clinical pharmacist and assistant; and work according to previously approved protocols and recipes.

Infusion pumps used in Bulgarian hospitals lack automatic dosing based on laboratory and physiological indicators. Doses are set manually by medical staff, who also monitor doses and infusion rates. The alarms integrated into the pumps have basic parameters and are not predictive. In our country, there is no centralised connectivity between infusion pumps and electronic health records. In home settings, pumps used for PN do not communicate with medical staff, and there is no telemedicine. There is also no integration with mobile applications for monitoring and control.

To better illustrate the current technological landscape, Table 4 presents a comparative overview of infusion systems used internationally and those currently applied in Bulgaria. The table highlights key differences in levels of technological development, particularly in the integration of artificial intelligence, automation capabilities, and clinical decision-support functions.

AI has not yet been widely implemented for parenteral nutrition, although there are projects and initiatives to digitalise and develop intelligent decision-support systems. Bulgarian research teams participate in international studies and publish work on the application of AI to optimise treatment and nutrition. To date, AI-controlled pumps are not widely used in Bulgarian hospitals; however, some new models donated in recent years support integration with hospital systems and intelligent alarms.

#### 3.3.6. Challenges for the Integration of Artificial Intelligence in Bulgaria

The implementation of artificial intelligence (AI) in healthcare systems in Bulgaria faces several important challenges that may limit its widespread adoption, particularly in specialised fields such as parenteral nutrition.

First, the digitalisation of hospital systems remains incomplete, which restricts the availability of structured electronic health data necessary for the development and application of AI-based models. Second, there is a lack of standardised protocols for data collection and management, resulting in inconsistencies in clinical datasets and limiting their usability for machine learning applications.

Another significant barrier is the limited funding available for the implementation of advanced health technologies and digital infrastructure. Investment in modern healthcare technologies remains essential for enabling the integration of AI tools into routine clinical practice.

Finally, the development of a clear regulatory framework for the protection of personal health data is crucial. Ensuring compliance with data protection regulations while enabling secure data sharing for research and clinical innovation remains a key challenge for Bulgaria’s healthcare system.

## 4. Discussion

Parenteral nutrition is life-saving, but it carries numerous risks of complications. To avoid complications, standards and formulas are developed to ensure precise dosage calculations [17,24]. The most appropriate solutions are personalised ones, based on sex, age, biometric indicators, and health status.

Evidence from global clinical practice demonstrates that the integration of artificial intelligence into parenteral nutrition management has substantial potential to improve patient outcomes by enabling individualised nutritional strategies, early prediction of metabolic and infectious complications, and real-time optimised clinical decision-making in real time [36].

The development of parenteral nutrition technologies and practices in Bulgaria over recent years reflects progress toward standardisation, wider availability of modern parenteral formulations, and increasing alignment with international clinical guidelines. Nevertheless, the adoption of advanced digital and intelligent systems that support personalised nutrition planning and continuous risk assessment remains limited.

This review highlights that the implementation of artificial intelligence in parenteral nutrition in Bulgaria presents both significant opportunities and notable challenges. Key barriers include limited availability and quality of clinical data, regulatory and ethical considerations, and the need for targeted education and training of healthcare professionals. At the same time, the potential benefits—such as reduced complication rates, shorter hospital stays, more efficient use of healthcare resources, and improved overall quality of care—underscore the strategic importance of artificial intelligence as a driver of innovation in parenteral nutrition [22,37].

Despite these promising developments, significant gaps persist in the literature. First, most current AI applications in clinical nutrition have mainly concentrated on neonatal intensive care settings, leaving adult PN management relatively underexplored. Second, many studies depend on retrospective datasets from single institutions, which restricts the generalisability of algorithmic recommendations across different healthcare systems. Third, there remains a lack of large prospective clinical trials assessing the real-world clinical outcomes of AI-assisted PN decision support systems.

Another important gap concerns the integration of AI solutions into national digital health infrastructures. Successful implementation requires interoperable electronic health record systems, standardised clinical data formats, and clearly defined data governance frameworks. Without these prerequisites, the translation of AI-driven innovations from research environments into routine clinical practice remains limited.

Furthermore, ethical considerations—including data privacy, algorithm transparency, and clinician oversight—must be carefully addressed [34]. Current research increasingly emphasises the importance of “human-in-the-loop” systems, in which AI tools support but do not replace clinical decision-making. This collaborative approach allows clinicians to maintain control while benefiting from AI-driven predictive analytics and precision recommendations [20].

## 5. Conclusions

The integration of artificial intelligence (AI) into parenteral nutrition (PN) offers promising opportunities to improve patient outcomes through personalised nutritional therapy and the early prediction of potential complications. The effective implementation of AI in PN requires a multidisciplinary approach that combines clinical expertise, technological innovation, and supportive healthcare policies. Such collaboration has the potential to sustainably enhance patient outcomes in parenteral nutrition in Bulgaria and to bring national clinical practice closer to leading international standards.

Although Bulgaria is still in the early stages of integrating AI and machine learning (ML) into parenteral nutrition, existing research and current initiatives indicate a positive trend toward further development in this field. The future of AI-supported PN in Bulgaria appears promising and is expected to significantly improve the quality and safety of patient care.

Nevertheless, the successful adoption of these technologies will require addressing several key challenges, including data standardisation, ethical considerations, and appropriate allocation of healthcare resources. In addition, further research is needed to generate robust scientific evidence on the benefits of implementing artificial intelligence in clinical practice. Such evidence would support informed decision-making and could provide a strong basis for encouraging institutional and governmental investment in the development and implementation of these innovative technologies.

## Figures and Tables

**Table 1:** Overview of Artificial Intelligence Applications in the Monitoring and Management of Parenteral Nutrition Complications.

Complication Focus	AI Methodology	Key Findings	Article
Metabolic complications	Machine Learning	Enhanced prediction capabilities for metabolic complications in PN patients.	Limketkai et al., 2021 [22]
Lipid instability	Machine Learning	Developed a model to predict lipid emulsion stability, addressing clinical safety concerns.	Shang et al., 2025 [17]
Malnutrition and infections	Generative AI	AI efficiently predicts nutritional outcomes and complications such as malnutrition.	Varayil et al., 2025 [23]
Malnutrition in cancer patients	Machine Learning	Improved decision support for individualized nutritional therapy.	Raphaeli and Singer, 2021 [24]
Enteral feeding intolerance	XGBoost Model	Early prediction model for enteral nutrition initiation in ICU patients.	Wang et al., 2023 [25]

**Table 2:** Applications of Artificial Intelligence in Parenteral Nutrition.

AI Function in Parenteral Nutrition	Description	Benefits for Patients and Clinical Staff
Automated calculation of PN requirements	Calculates individualised energy, protein, fluid, and micronutrient requirements based on body weight, age, organ function, laboratory parameters, and severity of illness [6,26]	Improved accuracy of PN prescriptions; reduced risk of underfeeding and overfeeding
PN formulation optimisation	Detects physicochemical incompatibilities and imbalances in glucose, amino acids, lipids, and electrolytes within PN admixtures [3]	Enhanced safety, stability, and metabolic tolerance of PN solutions
Predictive risk analysis	Uses clinical and laboratory data to predict metabolic complications such as hyperglycaemia, hypoglycaemia, electrolyte disturbances, and refeeding syndrome [1,8,11]	Early identification of complications and timely intervention
Real-time PN infusion monitoring	Monitors infusion rates, total volume, and duration; generates alerts when deviations from prescribed PN regimens occur [28]	Reduced infusion-related errors and decreased nursing workload
AI-based clinical decision support for PN	Provides evidence-based recommendations for complex PN scenarios (e.g., renal failure, hepatic dysfunction, sepsis, multi-organ failure) [6,24]	Clinical support and education are especially valuable for junior clinicians
Integration with ICU information systems (HIS/EMR)	Integrates PN management with electronic medical records, laboratory systems, and ICU nutrition protocols [25,26]	Coordinated, standardised, and traceable nutritional care

**Table 3:** Comparison between a standard infusion pump and an AI-based infusion pump [3,31,32,33].

Criterion	Standard Infusion Pump	AI-Based Infusion Pump
Dosing accuracy	High accuracy, but requires manual input and control	Automatically adapts dosing based on clinical data and patient condition
Infusion rate	Set and controlled manually by medical staff	Optimised in real time through continuous patient data analysis
Alarm system	Alerts triggered by mechanical issues (air in line, occlusion)	Intelligent, predictive alarms that warn before potential errors occur
Integration with electronic health records (EHR)	Limited or absent	Full integration with hospital information systems and automated dosing
Remote monitoring	Usually not supported	Enables real-time remote monitoring by healthcare professionals
Infusion setup	Manual configuration via buttons and menus	Automatic configuration based on protocols, weight, age, renal function, etc.
Automatic dose adjustment	Not available	Available—based on physiological responses and laboratory data

**Table 4:** Comparison of AI-Supported Infusion Systems Used Internationally and Infusion Systems Currently Used in Bulgaria.

Criteria	Leading Hospitals in the World	Hospitals in Bulgaria
AI functions	Yes—automatic dosing, predictive alarms, EHR connectivity	Partially—some have „drug libraries” and alarms, but without AI
Integration with electronic records	Fully integrated (EPIC, EHR, other hospital systems)	Limited—EHRs are used in some university hospitals, but are not connected to infusion systems
Remote monitoring	Yes—in real time, including mobile applications for TPN	No—monitoring is possible only on site
Used systems	Different types with integrated or non-integrated artificial intelligence	Different types
		All of them without AI
TPN	AI-controlled, adaptive nutrition related to blood sugar, kidney function, etc.	Manual adjustment by medical staff (dose, rate, volume)
Hospitals—examples	Mayo Clinic	Aleksandrovska University Hospital, Pirogov Hospital, St. Marina University Hospital, Burgas University Hospital, Pediatric Hospital “Prof. Ivan Mitev’’
	Virginia Oncology Associates	
Intelligent alarming systems	Yes—predictive (early detection of blockage, wrong dose, risk)	Mainly mechanical alarms—air, blockage, empty cartridge
Automatic dose adaptation	Yes—AI calculates the dose according to the patient’s condition	None—doses are entered manually, according to a doctor’s prescription
Investment in an infusion AI system	Millions of dollars/euro—supported by digital hospital platforms	Limited budget, donations, EU funds

## Data Availability

No new data were created or analyzed in this study. Data sharing is not applicable to this article.

## Abbreviations

The following abbreviations are used in this manuscript:

AI	Artificial Intelligent
ARDS	Acute Respiratory Distress Syndrome
EHRs	Electronic Health Records
PN	Parenteral Nutrition
TPN	Total Parenteral Nutrition
VIP	Volumetric Infusion Pump

## References

[1] Berlana D. (2022). Parenteral Nutrition Overview. Nutrients.

[2] Patel K.S., Noel P., Singh V.P. (2014). Potential influence of intravenous lipids on the outcomes of acute pancreatitis. Nutr. Clin. Pract..

[3] Ayers P., Adams S., Boullata J., Gervasio J., Holcombe B., Kraft M.D., Marshall N., Neal A., Sacks G., Seres D.S. (2014). A.S.P.E.N. parenteral nutrition safety consensus recommendations. J. Parenter. Enter. Nutr..

[4] ESPGHAN Guidelines. https://espghan.info/.

[5] Sobol Ż., Chiczewski R., Wątróbska-Świetlikowska D. (2025). The Modern Approach to Total Parenteral Nutrition: Multidirectional Therapy Perspectives with a Focus on the Physicochemical Stability of the Lipid Fraction. Nutrients.

[6] Singer P., Blaser A.R., Berger M.M., Alhazzani W., Calder P.C., Casaer M.P., Hiesmayr M., Mayer K., Montejo J.C., Pichard C. (2019). ESPEN guideline on clinical nutrition in the intensive care unit. Clin. Nutr..

[7] Lappas B.M., Patel D., Kumpf V., Adams D.W., Seidner D.L. (2018). Parenteral Nutrition: Indications, Access, and Complications. Gastroenterol. Clin. N. Am..

[8] Mahan L.K. (2016). Krause’s Food & the Nutrition Care Process.

[9] McClave S.A., Taylor B.E., Martindale R.G., Warren M.M., Johnson D.R., Braunschweig C., McCarthy M.S., Davanos E., Rice T.W., Cresci G.A. (2016). Guidelines for the Provision and Assessment of Nutrition Support Therapy in the Adult Critically Ill Patient. J. Parenter. Enter. Nutr..

[10] Hwang T.L., Chiang C.L., Wang P.N. (2001). Parenteral nutrition support after bone marrow transplantation: Comparison of total and partial parenteral nutrition during the early posttransplantation period. Nutrition.

[11] Galica L.A. (1997). Parenteral nutrition. Nurs. Clin. N. Am..

[12] Oguz S.S., Ergenekon E., Tümer L., Koç E., Turan O., Önal E., Türkyilmaz C., Atalay Y. (2011). A rare case of severe lactic acidosis in a preterm infant: Lack of thiamine during total parenteral nutrition. J. Pediatr. Endocrinol. Metab..

[13] Montalvo-Jave E.E., Zarraga J.L., Sarr M.G. (2007). Specific topics and complications of parenteral nutrition. Langenbeck’s Arch. Surg..

[14] Essentials of Total Parenteral Nutrition: A Review. https://search.emarefa.net/en/detail/BIM-292608-essentials-of-total-parenteral-nutrition-a-review.

[15] Wu X., Oniani D., Shao Z., Arciero P., Sivarajkumar S., Hilsman J., E Mohr A., Ibe S., Moharir M., Li L.-J. (2025). A Scoping Review of Artificial Intelligence for Precision Nutrition. Adv. Nutr. Int. Rev. J..

[16] Mehta S., Huey S.L., Fahim S.M., Sinha S., Rajagopalan K., Ahmed T., Knight R., Finkelstein J.L. (2025). Advances in Artificial Intelligence and Precision Nutrition Approaches to Improve Maternal and Child Health in Low Resource Settings. Nat. Commun..

[17] Shang Y., Wang X., Cheng Y., Qin W., Li P., Zhang L. (2025). Machine learning predicts lipid emulsion stability in parenteral nutrition using multi-laboratory literature data. Front. Nutr..

[18] Derakhshandara S., Franzoni V., Mezzetti D., Poggioni V. (2024). Machine Learning Decision Support for Macronutrients in Neonatal Parenteral Nutrition. IEEE Access.

[19] Yoon S.J., Kim D., Park S.H., Han J.H., Lim J., Shin J.E., Eun H.S., Lee S.M., Park M.S. (2023). Prediction of Postnatal Growth Failure in Very Low Birth Weight Infants Using a Machine Learning Model. Diagnostics.

[20] Phongpreecha T., Ghanem M., Reiss J.D., Oskotsky T.T., Mataraso S.J., De Francesco D., Reincke S.M., Espinosa C., Chung P., Ng T. (2025). AI-guided precision parenteral nutrition for neonatal intensive care units. Nat. Med..

[21] Yalçın N., Kaşıkcı M., Çelik H.T., Demirkan K., Yiğit Ş., Yurdakök M. (2023). Development and validation of machine learning-based clinical decision support tool for identifying malnutrition in NICU patients. Sci. Rep..

[22] Limketkai B.N., Mauldin K., Manitius N., Jalilian L., Salonen B.R. (2021). The Age of Artificial Intelligence: Use of Digital Technology in Clinical Nutrition. Curr. Surg. Rep..

[23] Varayil J.E., Bielinski S.J., Mundi M.S., Bonnes S.L., Salonen B.R., Hurt R.T. (2025). Artificial Intelligence in Clinical Nutrition: Bridging Data Analytics and Nutritional Care. Curr. Nutr. Rep..

[24] Raphaeli O., Singer P. (2021). Towards personalized nutritional treatment for malnutrition using machine learning-based screening tools. Clin. Nutr..

[25] Wang Y.-X., Li X.-L., Zhang L.-H., Li H.-N., Liu X.-M., Song W., Pang X.-F. (2023). Machine learning algorithms assist early evaluation of enteral nutrition in ICU patients. Front. Nutr..

[26] Mehta N.M., Skillman H.E., Irving S.Y., Coss-Bu J.A., Vermilyea S., Farrington E.A., McKeever L., Hall A.M., Goday P.S., Braunschweig C. (2017). Guidelines for the Provision and Assessment of Nutrition Support Therapy in the Pediatric Critically Ill Patient: Society of Critical Care Medicine and American Society for Parenteral and Enteral Nutrition. J. Parenter. Enter. Nutr..

[27] Briassoulis G., Briassouli E. (2025). AI-Enabled Precision Nutrition in the ICU: A Narrative Review and Implementation Roadmap. Nutrients.

[28] Hadweh P., Niset A., Salvagno M., Al Barajraji M., El Hadwe S., Taccone F.S., Barrit S. (2025). Machine Learning and Artificial Intelligence in Intensive Care Medicine: Critical Recalibrations from Rule-Based Systems to Frontier Models. J. Clin. Med..

[29] Singer P., Robinson E., Raphaeli O. (2023). The future of artificial intelligence in clinical nutrition. Curr. Opin. Clin. Nutr. Metab. Care.

[30] Boullata J.I., Gilbert K., Sacks G., Labossiere R.J., Crill C., Goday P., Kumpf V.J., Mattox T.W., Plogsted S., Holcombe B. (2014). A.S.P.E.N. clinical guidelines: Parenteral nutrition ordering, order review, compounding, labeling, and dispensing. J. Parenter. Enter. Nutr..

[31] Center for Drug Evaluation and Research (2025). Compounding and the FDA: Questions and Answers.

[32] Raimbault M., Thibault M., Lebel D., Bussières J.-F. (2012). Automated Compounding of Parenteral Nutrition for Pediatric Patients: Characterization of Workload and Costs. J. Pediatr. Pharmacol. Ther..

[33] B. Braun Standard Product Catalog|B. Braun. https://catalogs.bbraun.com/en-01/c/PRODUCTS/b-braun-standard-product-catalog.

[34] Ballhausen H., Corradini S., Belka C., Bogdanov D., Boldrini L., Bono F., Goelz C., Landry G., Panza G., Parodi K. (2024). Privacy-friendly evaluation of patient data with secure multiparty computation in a European pilot study. npj Digit. Med..

[35] Republic of Bulgaria (2009). Ordinance No. 10 of 2009 on the Conditions, Procedure, Mechanism and Criteria for Payment by the National Health Insurance Fund of Medicinal Products, Medical Devices, Dietary Foods for Special Medical Purposes and of Auxiliary Aids, Devices, Equipment and Medical Devices for People with Disabilities, Negotiation of Discounts and Reimbursement of Excess Funds Under a Mechanism Guaranteeing Predictability and Sustainability of the NHIF Budget (State Gazette No. 24 of 31 March 2009, as Amended).

[36] Peng D., Phongpreecha T., Aghaeepour N. (2025). Artificial intelligence-guided nutritional therapy in the ICU. Curr. Opin. Clin. Nutr. Metab. Care.

[37] Lee S.M., Park J. (2025). Risk factors for complications associated with peripherally inserted central venous catheters for parenteral nutrition: Machine learning and survival analysis based on deep learning. Clin. Nutr..

